# Altered microRNA profiles in plasma exosomes from mesial temporal lobe epilepsy with hippocampal sclerosis

**DOI:** 10.18632/oncotarget.13744

**Published:** 2016-12-01

**Authors:** Shaofeng Yan, Hua Zhang, Wenyan Xie, Fangang Meng, Kai Zhang, Yin Jiang, Xin Zhang, Jianguo Zhang

**Affiliations:** ^1^ Department of Neurosurgery, Beijing Tiantan Hospital, Capital Medical University, Beijing, China; ^2^ Department of Clinical Laboratory, Qian Fo Shan Hospital of Shandong Province, Jinan, Shandong Province, China; ^3^ Department of Functional Neurosurgery, Beijing Neurosurgical Institute, Capital Medical University, Beijing, China

**Keywords:** exosome, microRNA, epilepsy, hippocampal sclerosis

## Abstract

Mesial temporal lobe epilepsy with hippocampal sclerosis (mTLE-HS) is the most common type of focal epilepsy. The present study aimed to explore the expression and functions of exosomal microRNAs in mTLE-HS. A total of 50 microRNAs were found to be differentially expressed in mTLE-HS compared with healthy controls. Among them, 2 were increased and 48 were decreased. The 6 significant differentially expressed candidate microRNAs (miR-3613-5p, miR-4668-5p, miR-8071, miR-197-5p, miR-4322, and miR-6781-5p ) in exosome were validated. The bioinformatics analysis showed that the potential target genes of these microRNAs were involved in biological processes, molecular functions, and cellular components. Similarly, these microRNAs also affected axon guidance, pathways in cancer, regulation of the actin cytoskeleton, focal adhesion, the calcium signaling pathway, the MAPK signaling pathway, and the PI3K-Akt signaling pathway. Among 6 candidate microRNAs, miR-8071 had the best diagnostic value for mTLE-HS with 83.33% sensitivity and 96.67% specificity, and was associated with seizure severity. This study indicated that exosomal microRNAs, may be regulators for the seizure development in mTLE-HS, and can be used as potential therapeutic targets and biomarker for diagnosis in mTLE-HS.

## INTRODUCTION

Epilepsy, estimated to affect about 65 million individuals worldwide [1], is a serious neurological disorder syndrome of refractory seizures resulting in devastating effects on patients [2]. Mesial temporal lobe epilepsy with hippocampal sclerosis (mTLE-HS) is the most common type of focal epilepsy, which is characterized by spontaneous recurrent seizures, neuronal cell loss, and mossy fibers sprouting in the hippocampus [3–5]. Status epilepticus can impair brain function and memory, which is a threat to the lives of patients [6]. At the present, the exact underlying molecular mechanisms of mTLE-HS are still unclear. In addition, accurate diagnosis and timely treatment can achieve good therapeutic effect for epilepsy. Therefore, understanding the pathogenesis of mTLE-HS and improvement of early diagnosis and effective treatments are required.

Exosomes, a specific subtype of secreted membrane vesicles that are approximately 30–100 nm in size, are involved in cell-to-cell communication and targeting cells by transferring exosomal molecules including proteins, mRNAs, and microRNAs (miRNAs) [7, 8]. Exosomes are widely distributed in the blood, urine, milk, and other bodily fluids [9]. Exosomal miRNAs have been identified, both in physiological and disease conditions [9]. Furthermore, the quantity and composition of exosomal miRNAs are different between patients and healthy individuals [10]. Exosomal miRNAs are involved in the origin and progress of diseases, such as viral infection, cancer, and central nervous system degenerative diseases [11, 12]. Exosomal miRNAs may serve as valuable noninvasive biomarkers for the diagnosis and prognosis of certain diseases, including glioma, Alzheimer's disease (AD), and Parkinson's disease (PD) [13, 14].

There is growing evidence supporting miRNAs changes in the pathophysiology of epilepsy [1]. However, exosomal miRNAs in the plasma of mTLE-HS patients have not yet been investigated. The primary goal of this study was to characterize differences in exosomal miRNAs in plasma from mTLE-HS patients and to further explore their biological functions and clinical significance.

## RESULTS

### MiRNAs were differentially expressed from plasma exosomes in mTLE-HS patients compared with healthy controls

**Figure 1:**
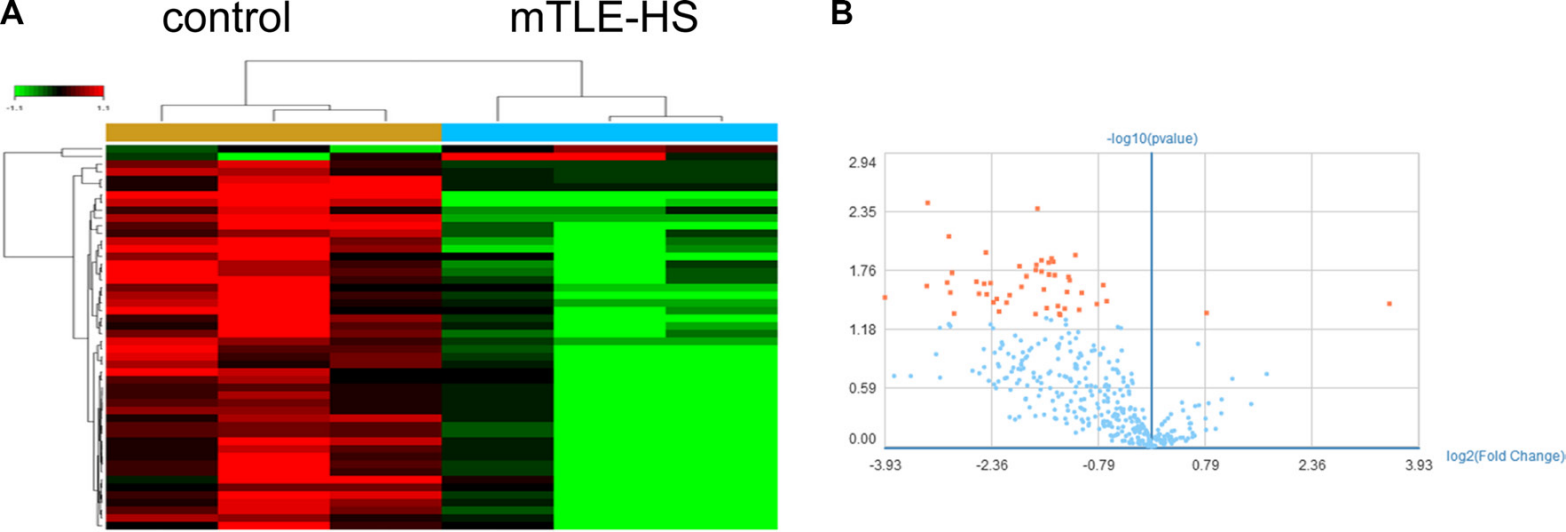
Microarray assay of exosomal miRNAs differentially expressed in the plasma from mTLE-HS patients and healthy controls (**A**) Heat map showing the differencial expression of exosomal miRNAs in mTLE-HS patients compared to normal healthy controls. Each column represents an individual sample and each row represents a single miRNA. Expression level of each miRNA in a single sample is depicted according to the color scale. Red represents high expression, whereas green represents low expression. (**B**) The volcano plot shows the relation between the logarithm of the *p*-values on the x-axis and the log fold change between mTLE-HS patients and healthy controls on the y-axis. The vertical line marks the border between mTLE-HS patients and healthy control results. Red represents miRNAs with expression changes of more than 1.2 fold from the remaining miRNAs, while blue represents miRNAs with expression changes of less than 1.2 fold from the remaining miRNAs.

First, miRNAs profiles of plasma exosomes from three mTLE-HS patients and gender and age matched three healthy controls were investigated by using Affymetrix miRNA 4.0 Arrays (Supplementary Table S1). The microarray results showed the differential expression of 50 exosomal miRNAs in mTLE-HS patients compared to those in healthy controls. Among these, we found that 2 miRNAs were significantly increased and 48 miRNAs were significantly decreased (fold change > 1.2; *P* < 0.05) in mTLE-HS patients when compared to healthy controls. Group specific signal intensities of the exosomal miRNAs profile and the volcano plot for the differentially expressed miRNAs between mTLE-HS patients and control healthy are shown in Figure 1A and Figure 1B, and the information of the 50 miRNAs is listed in Table 1 with fold changes and *P* values.

**Table 1:** 50 differentially expressed exosomal miRNAs in plasma in mTLE-HS versus controls

microRNA	Fold-change	Style	*p*-value	rank
hsa-miR-4668-5p	−9.854432	down	0.003634	1
has-miR-4322	−3.210969	down	0.004135	2
hsa-miR-3613-3p	−7.927916	down	0.007895	3
hsa-miR-8071	−5.435439	down	0.011404	4
hsa-miR-6781-5p	−2.184829	down	0.012155	5
hsa-miR-1306-3p	−2.785553	down	0.013158	6
hsa-miR-197-5p	−3.079638	down	0.013659	7
hsa-miR-3648	−2.732113	down	0.01416	8
hsa-miR-6790-5p	−2.870511	down	0.014411	9
hsa-miR-6510-5p	−3.247119	down	0.015163	10
hsa-miR-3180-3p	−3.867019	down	0.015664	11
hsa-miR-483-5p	−3.268119	down	0.017168	12
hsa-miR-4481	−3.081376	down	0.017669	13
hsa-miR-1275	−7.679397	down	0.01817	14
hsa-miR-671-5p	−2.858979	down	0.018922	15
hsa-miR-6856-5p	−2.697207	down	0.019173	16
hsa-miR-6808-5p	−3.600177	down	0.019674	17
hsa-miR-4417	−2.340955	down	0.019925	18
hsa-miR-3162-5p	−2.315872	down	0.021429	19
hsa-miR-4253	−5.996269	down	0.02218	20
hsa-miR-4649-5p	−8.082261	down	0.022682	21
hsa-miR-6782-5p	−5.188365	down	0.022932	22
hsa-miR-6824-5p	−5.532658	down	0.023183	23
hsa-miR-3149	−1.644625	down	0.023935	24
hsa-miR-6716-5p	−9.950737	down	0.024937	25
hsa-miR-5196-5p	−3.784796	down	0.026441	26
hsa-miR-150-3p	−3.011115	down	0.028195	27
hsa-miR-1183	−2.37409	down	0.028195	28
hsa-miR-4689	−7.814024	down	0.028446	29
hsa-miR-6124	−2.04976	down	0.028697	30
hsa-miR-7150	−5.826567	down	0.029198	31
hsa-miR-939-5p	−5.391403	down	0.029669	32
hsa-miR-6819-5p	−4.27274	down	0.030201	33
hsa-miR-4443	−15.229739	down	0.031955	34
hsa-miR-6891-5p	−4.862422	down	0.032957	35
hsa-miR-6827-5p	−1.589722	down	0.034712	36
hsa-miR-6802-5p	−5.038429	down	0.05714	37
hsa-miR-1909-3p	−4.403976	down	0.035965	38
hsa-miR-3613-5p	11.28126	up	0.036967	39
hsa-miR-4721	−1.753045	down	0.037218	40
hsa-miR-4462	−2.61099	down	0.038972	41
hsa-miR-7114-5p	−2.929565	down	0.040727	42
hsa-miR-4656	−2.432757	down	0.041479	43
hsa-miR-6871-5p	−2.100326	down	0.042481	44
hsa-miR-6794-5p	−4.753529	down	0.044236	45
hsa-miR-6511b-5p	1.744463	up	0.045739	46
hsa-miR-6798-5p	−7.536286	down	0.046241	47
hsa-miR-4298	−2.570175	down	0.046742	48
hsa-miR-6799-5p	−3.278097	down	0.046992	49
hsa-miR-1307-3p	−2.54687	down	0.047744	50

### QRT-PCR verification of exosomal miRNAs expression

**Figure 2:**
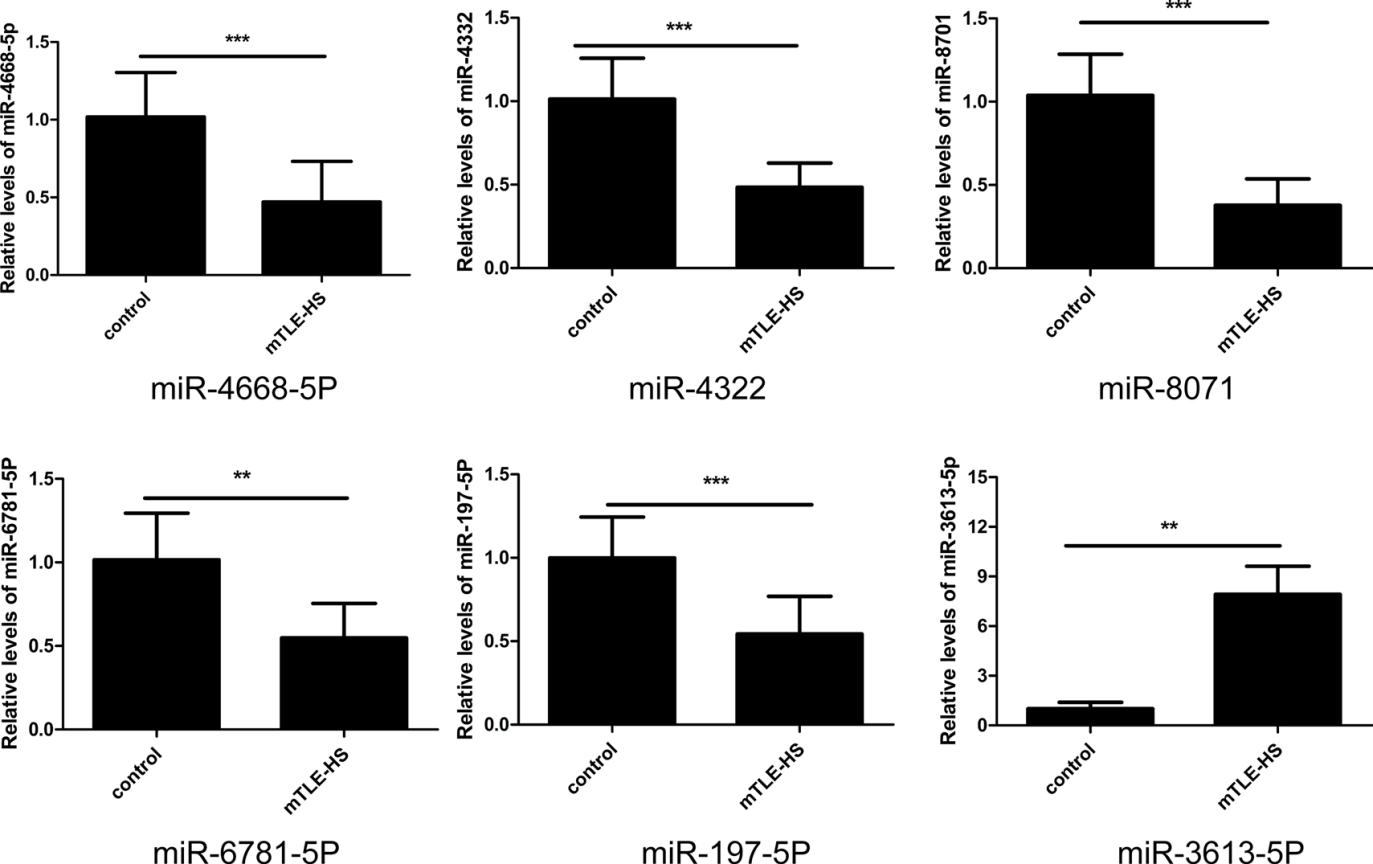
Validation of differential exosomal miRNAs by qRT-PCR in mTLE-HS and healthy control groups The results showed the relative expression levels of miR-4668-5P, miR-4322, miR-8071, miR-6781, miR-197-5p, and miR-3613-5p in 40 mTLE-HS patients and 40 healthy controls. Cel-miR-39-3p was used as a reference gene. *p < 0.05, **p < 0.01, ***p < 0.001.

To validate the accuracy of the microarray based miRNA measurements, quantitative reverse transcriptase polymerase chain reaction (qRT-PCR) assays was performed. Expression levels of the 6 miRNAs selected from the miRNAs chip were determined in a cohort of 40 mTLE-HS patients and 40 healthy controls (Supplementary Table S1 and Supplementary Table S2). Among these selected miRNAs, the results showed that expression levels of exosomal miRNAs (miR-4668-5P, miR-4322, miR-8071, miR-6781-5P, and miR-197-5p) were significantly decreased in the mTLE-HS patients (Figure 2), and the expression lever of (miR-3613-5p) was significantly increased (Figure 2). The qRT-PCR results were consistent with the microarray data. The results suggested that the data obtained from the miRNA microarrays accurately reflected the exosomal miRNA expression levels in the plasma from the mTLE-HS patients compared with the healthy controls.

### Bioinformatics prediction revealed the role of exosomal miRNAs in mTLE-HS

**Figure 3:**
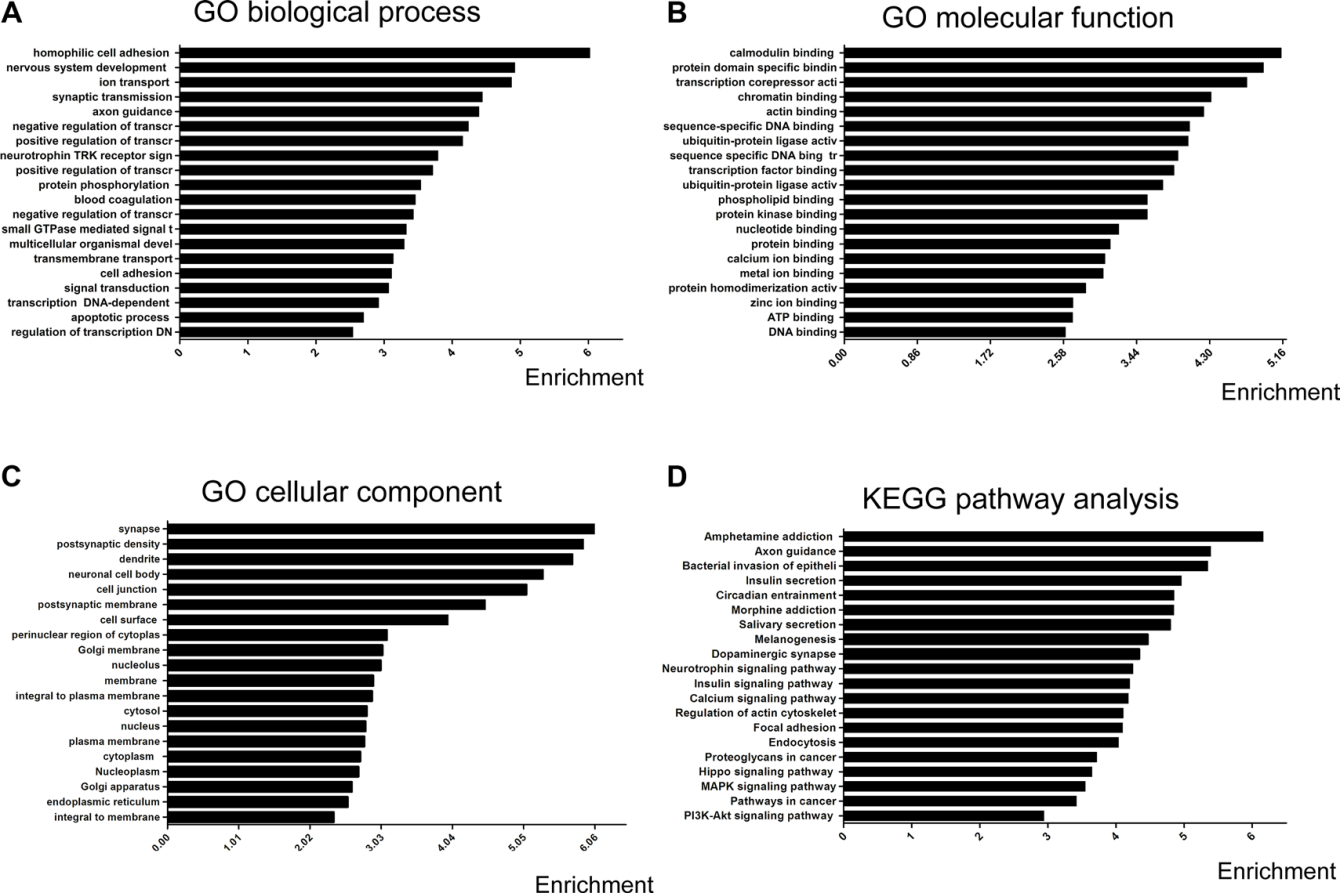
Prediction of functions and signal pathways of differential exosomal miRNAs GO categories and distribution for the predicted miRNA targets related differential miRNAs The GO terms and signal pathways terms were sorted by enrichment scores of genes in ascending order from top to bottom. (**A**) Biological processes are enriched in signaling transduction, regulation of transcription, multicellular organismal development, cell adhesion, and ion transport. (**B**) Cellular components are enriched in the nucleus, cytoplasm, and membrane. (**C**) Molecular functions are enriched in protein binding, metal ion binding, and zinc ion binding (**D**) Pathway analysis based on potential target genes of differential miRNAs The results show the significant pathways targeted by differential miRNAs. which are rich in axon guidance, pathways in cancer, regulation of actin cytoskeleton, focal adhesion, the calcium signaling pathway, the MAPK signaling pathway, and the PI3K-Akt signaling pathway.

**Figure 4:**
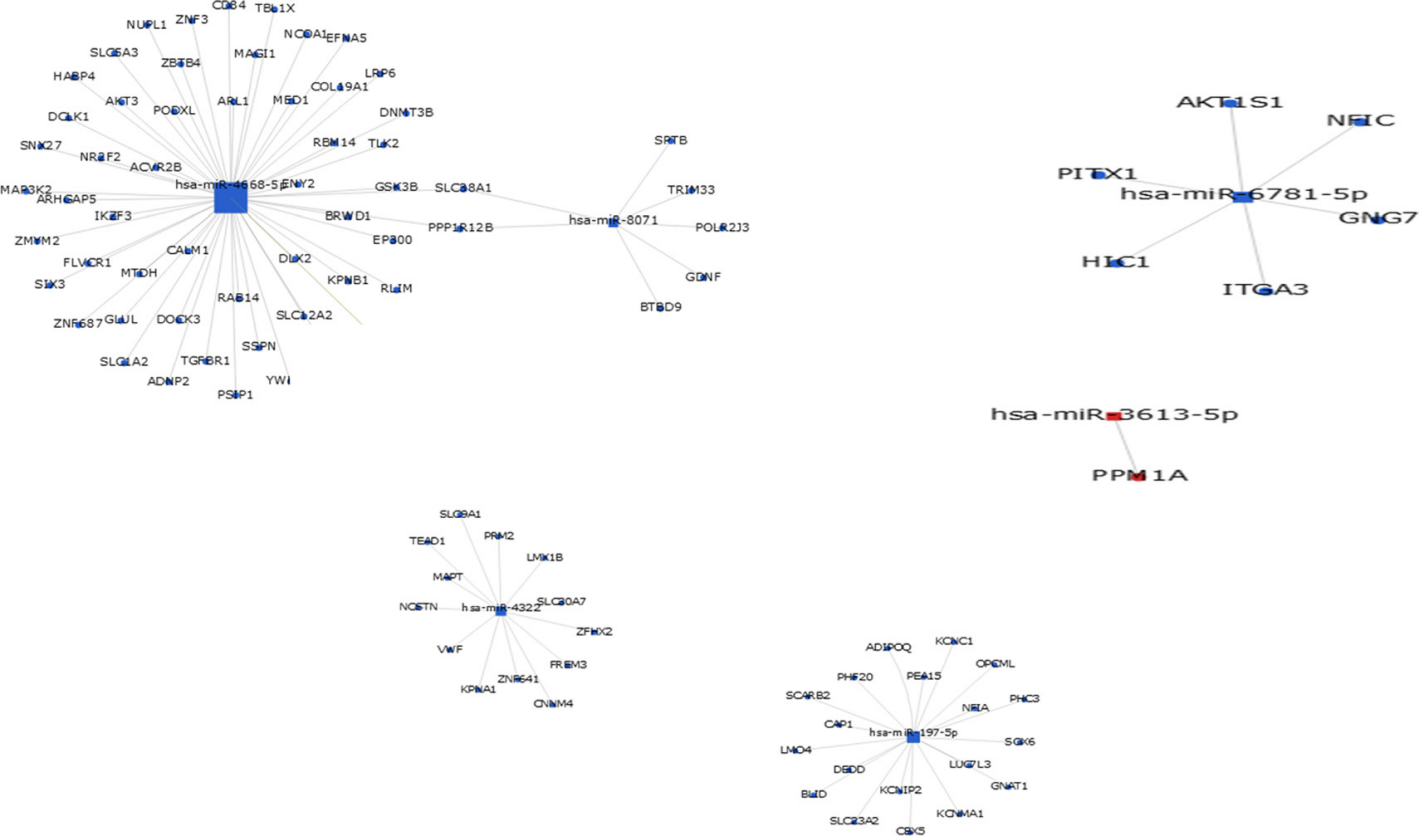
MiRNA-gene network The gray circle represents gene (mRNA), the square represents miRNA (the red marker represents up-regulated miRNA and blue marker represents down-regulated miRNA). The relationship between the miRNA and gene is represented by a green line.

To assess the potential biological function of the differentially expressed miRNAs, GO annotations and KEGG pathway enrichment analysis were performed, based on three available databases (microRNA, miRBase, and TargetScan). The primary GO terms of the predicted target genes for differentially expressed miRNAs mainly included biological processes, cellular components, and molecular functions. Gene-term enrichment analysis revealed that most predicted target genes were involved in homophilic cell adhesion, synaptic transmission, signal transduction, cell adhesion, negative regulation of transcription from RNA polymer, positive regulation of transcription from RNA polymeras, protein phosphorylation, apoptotic processes (GO biological process) (Figure 3A), calmodulin binding, protein domain specific binding, chromatin binding, transcription corepressor activity, actin binding (Figure 3B), dendrites, synapses, postsynaptic density, postsynaptic membrane, and cell surface (GO cellular component) (Figure 3C). The KEGG pathway analysis showed that the predicted target genes were involved in biological pathways including axon guidance, pathways in cancer, regulation of the actin cytoskeleton, focal adhesion, the calcium signaling pathway, the MAPK signaling pathway, and the PI3K-Akt signaling pathway (Figure 3D). To further illustrate the relationship between the overlap of miRNAs and their targeted genes, the miRNA-mRNA network was generated (Figure 4), based on the GO and the KEGG predicted data (see above). The results suggested that differentially expressed exosomal miRNAs were involved in a wide variety of physiological processes in the development of mTLE-HS.

### Evaluation of exosomal miRNAs as potential diagnostic markers

**Figure 5:**
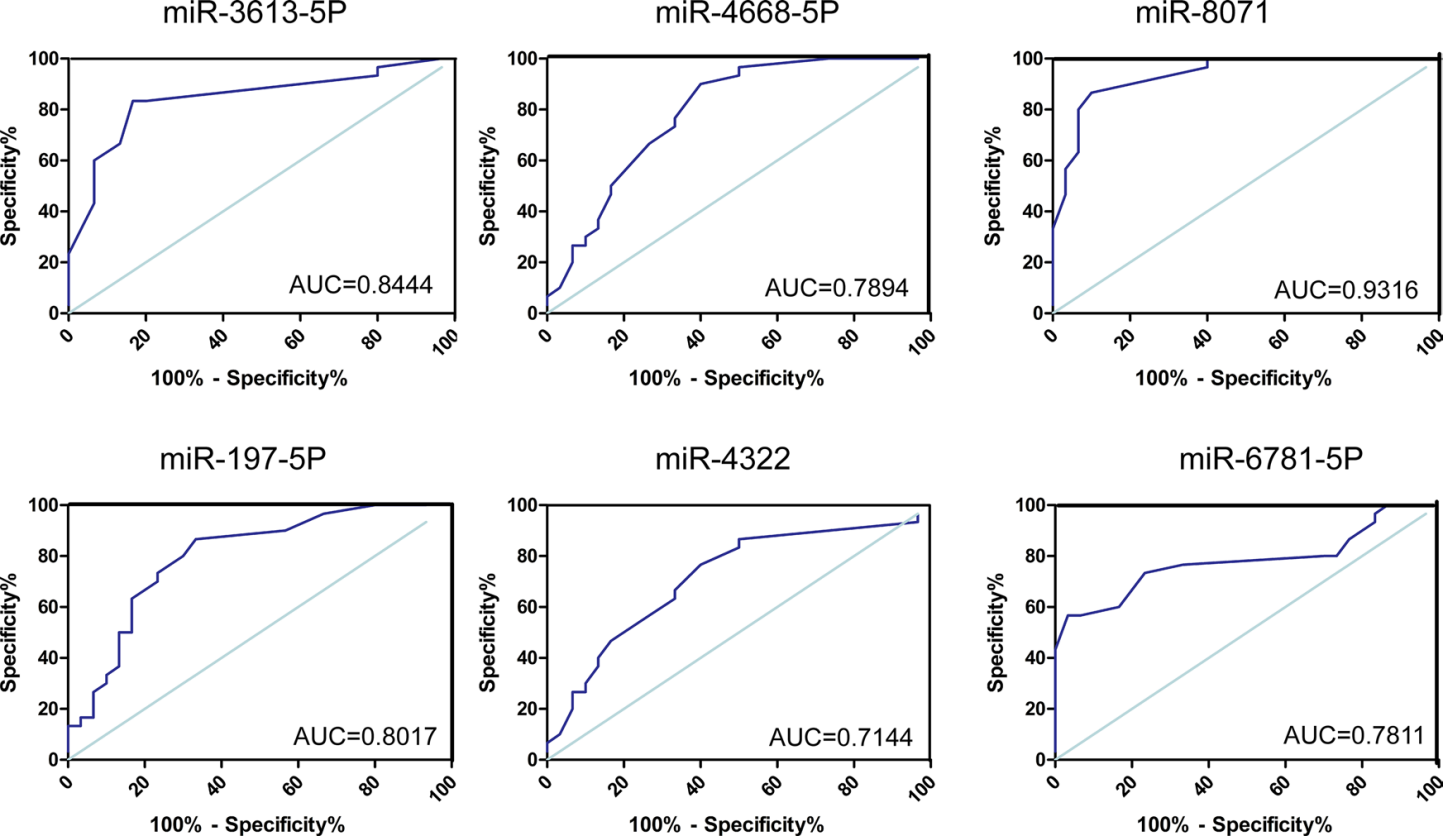
ROC curves for miRNAs that are significantly different in mTLE-HS patients as compared to healthy controls ROC curves for exosomal miR-4668-5P, miR-4322, miR-8071, miR-6781, miR-197-5p, and miR-3613-5p were used to discriminated mTLE-HS patients and healthy controls and the area under the curve (AUC) was used to evaluate the level of discrimination.

To evaluate the value of exosomal miRNA levels in discriminating cases of mTLE-HS from healthy controls, ROC curve analysis was performed. Comparing the mTLE-HS and control groups, as shown in Figure 5 and Table 2, the ROC curve areas for miR-3613-5p, miR-4668-5p, miR-8071, and miR-197-5p were found to be 0.8444 (95% CI, 0.7402–0.9487), 0.7894 (95% CI, 0.6732–0.9057), 0.9316 (95% CI, 0.8768–0.9955), and 0.8017 (0.6881–0.9152 (Figure 5), respectively. The AUC for the other two miRNAs (miR-4322 and miR- 6781-5p) were > 0.7 but did not reach statistical significance (Figure 5 and Table 2). More importantly, the results showed that miR-8071 was the most valuable biomarker for discriminating patients from healthy individuals, and the best cutoff of miR-8071 was 0.4500 with sensitivity and specificity of 83.33% and 96.67%, respectively. These data demonstrated that exosomal miRNAs in plasma are reliable diagnostic markers for mTLE-HS.

**Table 2:** Area under the receiver operating characteristic curve (AUC), 95% confidence interval (CI), and *P* values of the differentially expressed microRNAs

Exomal- miRNAs	AUC	95% CI	*P* value
miR-3613-5p	0.8444	0.7402–0.9487	0.0121
miR-4668-5P	0.7894	0.6732–0.9057	0.0363
miR-4322	0.7144	0.5817–0.8472	0.0634
miR-8071	0.9316	0.8768–0.9955	0.0286
miR-197-5P	0.8017	0.6881–0.9152	0.0052
miR-6781-5P	0.7811	0.6585–0.9037	0.0743

### Correlation between exosomal miRNAs and clinical characteristics

The above results suggested that exosomal miRNAs are good diagnostic biomarkers of mTLE-HS. We further analyzed the association between exosomal miR-8071 expression and clinicopathological characteristics in mTLE-HS patients. The expression levels of exosomal miR-8071 were categorized as low or high in relation to the median value. Our results demonstrated that there was no significant correlation between exosomal miR-8071 expression and clinicopathological features, such as age, gender, family history of epilepsy, seizure time, laterality of the epileptogenic zone, AED therapy at the last clinic visit, sampling time from the last seizure, and comorbid conditions (Table 3). However, we found that the exosomal miR-8071 level was significantly associated with disease duration or seizure frequency (Table 3). These results indicated that exosomal miRNAs were involved in the epileptogenesis of mTLE-HS.

**Table 3:** Demographic and clinical data of patients with mTLE-HS

Characteristic	Exosmal miR-8071 expression		*P*
	High	Low	
Male: Female	12:8	13:7	0.1257
Age (range)	26.21 years (11–38)	28.32 years (15–49)	0.0630
Family history of epilepsy	1 (5%)	3 (17%)	0.0720
Course of epilepsy (range)	10.05 years (1–28)	17.41 years (1–26)	0.0073*
Onset of seizure (range)	18.06 years (2–30)	12.38 years (0.5–29)	0.0661
Seizure frequency (range)(months)	28.60 (1–300)	81.20 (1–330)	0.0316*
Seizure time (range)	1.55 min (5 s–5 min)	2.20 min (2 s–10 min)	0.0680
Laterality of epileptogenic zone			
Right	8 (40%)	7 (35%)	0.2926
Left	10 (50%)	13 (65%)	
Bilateral	2 (10%)	0 (0%)	
Sampling time from the last seizure (range)	5.80 (1–28)	6.52 (3–30)	0.0875
AED therapy at the last clinic visit			
Valproic acid	15	10	0.8600
Carbamazepine	18	17	
Levetirasetam	8	12	
Oxcarbazepine	12	14	
Lamotrigine	6	4	
Topiramate	3	3	
Others	2	3	
Comorbid conditions			
Hypertension	2	1	0.3910
Diabetes	1	1	
Hyperlipidemia	1	1	
Others	0	0	

## DISCUSSION

Recently, numerous studies have shown that exosomal miRNAs were involved in pathophysiology of diseases, especially in cancer, neurodegenerative diseases, and infection [8, 12, 13]. However, the previous studies of mTLE-HS have focused on animals or circulating miRNAs, and were seldom related to exosomal miRNAs [1, 15]. In the present study, our results showed a differential exosomal miRNAs expression profile in plasma from mTLE-HS patients compared with healthy controls.

Epileptogenesis is thought to be involved in several processes, such as neuronal loss, gliosis, gene regulation, axonal sprouting, inflammation, and neurogenesis [16–18]. MiRNAs often suppressed expression of multiple proteins by inhibiting translation or targeting mRNAs at 3′UTR for degradation [19]. According the previous reports, current bioinformatics tools for miRNA-target analysis were widely used in studying association between miRNAs and diseases, including cancer, inflammation, neurodegenerative diseases and epilepsy [12, 13, 15, 20]. These studies indicated these miRNA target prediction tools were effective and accuracy for miRNA target prediction. We further investigated the possible functions of miRNAs through GO terms and KEGG pathway annotation, based on bioinformatics tools. Our results suggested that cell adherence, the cell cycle, and apoptosis were significant GO categories affected by these dysregulated miRNAs. More importantly, these biological processes may also contribute to epileptogenesis. For example, as previously reported, cell apoptosis can cause hippocampal neuronal loss after epileptic seizures [15].

The KEGG is widely used for analysis of various types of molecular biological data in order to discover signaling pathways [21]. Our results showed that many signaling pathways were, as identified by KEGG pathway annotation, affected by these dysregulated microRNAs, such as axon guidance, pathways in cancer, regulation of the actin cytoskeleton, focal adhesion, the calcium signaling pathway, the MAPK signaling pathway, and the PI3K-Akt signaling pathway. As previously reported, axon guidance has been linked to epilepsy, with the sprouting of mossy fibers (the axons of granule cells) being the best characterization of axonal reorganization in TLE [22]. The neurotrophin receptor-interacting factor (NRIF) mediates apoptotic signaling via p75(NTR) in hippocampal neurons *in vitro* and *in vivo* [23]. The PI3K/Akt and ERK1/2 signaling pathways mediate the EPO-modulated calcium influx in KA-induced epilepsy [24]. To further investigate the function of differentially expressed exosomal miRNAs, the miRNA-mRNA regulatory network was analyzed by bioinformatic observations, and then the main targets of miRNAs were outlined (shown in Figure 5). These results suggested that exosomal miRNAs may have regulatory effects on the pathogenesis of epilepsy by affecting lots of biological progress and signaling pathways as mentioned above.

Currently, the diagnosis of epilepsy mainly depends on the complaint, CT, MRI, and EEG, which can be inaccurate and costly. Exploring biomarkers which could distinguish between epileptic and healthy individual is required. Compared to mRNAs and even proteins, miRNAs are in a relatively stable form, which makes miRNAs potential candidates as noninvasive biomarkers [25]. Some preclinical studies on epilepsy offer the exciting possibility of miRNAs acting as biomarkers for disease diagnosis, stratification, and monitoring [1]. In our study, in order to evaluate the efficiency of these miRNAs for diagnosing mTLE-HS, ROC curves were constructed for each miRNA. ROC curves for four miRNAs (miR-3613-5p, miR-4668-5p, miR-8071, and miR-197-5p) showed good ability to efficiently distinguish mTLE-HS patients from healthy control, with an AUC that ranged from 0.6482 to 0.9955. These results indicated that exosomal miRNAs could be used as better biomarker of diagnosis of mTLE-HS. It would be interesting to determine the relation between exosomal miRNAs and clinical parameters. More importantly, we proved that exosomal miR-8071 expression was significantly associated with seizure severity of patients with mTLE-HS. Thus, these results indicated that exosomal miRNAs were involved in the progression of mTLE-HS. However, we need to further study the function mechanism of exosomal miRNAs on epileptogenesis. In addition, interestingly enough, the exosome could be released from kinds of organs, including muscles, liver, blood cells, and brain tissues [26–29]. Due to the complexity of mTLE-HS in pathogenesis and treatment, now, it is not clear that which organs exosomal miRNAs are from. This requires that we continue making great efforts and studying further.

In conclusion, the present study is the first to evaluate the plasma miRNA expression pattern in mTLE-HS patients by microarray-based miRNA analysis. Functional bioinformatics analysis demonstrated that the target genes regulated by these miRNAs were involved in several biological processes and signaling pathways. Expression levels of exosomal miRNAs (miR-3613-5p, miR-4668-5p, miR-8071, and miR-197-5p) showed good ability to efficiently distinguish mTLE-HS patients from healthy controls. The study of these miRNAs may provide a clearer understanding on the pathogenesis of mTLE-HS and indicate that exosomal miRNAs may be used as potential therapeutic targets and diagnostic biomarkers for mTLE-HS.

## MATERIALS AND METHODS

### Plasma collection

In this study, 40 patients diagnosed with mTLE-HS and gender and age matched 40 healthy volunteers were recruited in the Tiantan Hospital (Beijing, China). 40 patients were referred for surgical resection of the temporal lobe by an epileptologist following extensive evaluation including neurological assessment, video EEG recording, and MRI neuroimaging [30, 31]. Each patient was determined to have medically intractable epilepsy with a history of recurring seizures, and hippocampal sclerosis was confirmed by postoperative pathology. A 5 ml sample of venous blood was collected from each patient diagnosed mTLE-HS, and a 5 ml sample of venous blood from gender and age matched healthy volunteers as the control group. Blood samples were drawn into EDTA-containing tubes and the plasma was immediately separated using a centrifuge at room temperature (centrifugation at 2,500 × g for 10 min). The supernatant was then transferred into RNase-free EP tubes and stored at −80°C until use. This study was approved by the Research Ethics Committee of Tiantan Hospital (Beijing, China) and informed consent was given by each subject.

### Isolation of exosomes

Exosomes were isolated from the plasma of the patient and healthy control groups by exoquick precipitation (RIBO, Guangzhou, China) according manufacturer's instructions [32]. After the cold plasma was thawed at 4°C, the plasma was subjected to successive centrifugations of 2,000 × g (20 min) and 10,000 × g (20 min). Then, the plasma was transferred into new EP tubes and the mixed reagent was added to the plasma and placed at 4°C for 30 min. Exosomes pellets were centrifuged to remove the supernatant at 10,000g for 30 min and the exosomes were identified by transmission electron microscopy (TEM) or Western blot analysis (not shown).

### RNA processing and miRNA profiling

Exosomal RNA was extracted using trizol reagent (Life Technologies, Carlsbad, CA, USA) according to the manufacturer's instructions. RNA quality and quantity was measured according to the OD260/280 using a Nanodrop ND-1000 system (Thermo Fisher Scientific, Waltham, USA). The exosomes from plasma of three mTLE-HS patients and three healthy volunteers were investigated by miRNA microarray analysis. Exosomal miRNAs were extended and hybridized with fluorescence labeled with biotin dyes on a Gene Chip miRNA 4.0 Array (Affymetrix, Cleveland, OH, USA). Following hybridization, the images were digitized and analyzed using a laser scanner interfaced with ArrayPro image analysis software (Media Cybernetics, Silver Spring, MD, USA). Data were analyzed by first subtracting the background and then the signals were normalized using a LOWESS filter (Locally-weighted Regression) [33] The differentially expressed miRNAs were defined using the ratio of detected signals log2-fold changes [log2(mTLE-HS/control)] and the Student's *t-test* was used to calculate *P* values. Those with a log2 ratio > 1.2. or ≤ −1.2 and *P* values < 0.05 were considered as differentially expressed miRNAs. Cluster analysis based on the relative expression levels of miRNAs was also carried out.

### QPCR miRNA assay for individual miRNAs

To validate the initial results of the miRNA microarray assay, qRT-PCR analyses were carried out using the Thermal Cycler Dice (TAKALA, Hercules, CA). Five down-regulated miRNAs (miR-4668-5P, miR-4322, miR-8071, miR-6781, and miR-197-5p) and one up-regulated (miR-3613-5p) were selected for subsequent qRT-PCR confirmation. The primers of miRNAs were purchased from RIBO (Guangzhou, China). Real-time PCR for miRNAs was performed according to previous reports in triplicate for each sample [20]. The relative amount of each miRNA was normalized against cel-miR-39-3p by the 2 ^−ΔΔCt^ method.

### Bioinformatics analysis of differentially expressed exosomal miRNAs

The target genes of differentially exosomal miRNAs were predicted by TargetScan (http://www.targetscan.org/) microRNA (http:/www.microrna.org/microrna/home.do) and miRBase (http://www.mirbase.org) [20]. The Gene Ontology (GO) and Kyoto Encyclopedia of Genes and Genomes (KEGG) database analyses were performed by a DAVID online analysis tool (http://david.abcc.ncifcrf.gov/) [34, 35]. Fisher's two-side exact test and Chi-square test were used to classify the GO categories and KEGG pathway categories, and the false discovery rate (FDR) was also calculated to correct the *P* values. We chose only GOs and the enriched pathways that had a *P value* of < 0.05 and a FDR of < 0.05. The relationship between miRNAs and genes was counted by their differential expression values and according to their interactions in the Sanger miRNA database. The miRNA-mRNA networks were generated by Gene-Cloud of Biotechnology Information (GCBI) (https://www.gcbi.com.cn/gclib/html/index).

### Statistical analysis

All statistical analysis was performed using the SPSS 17.0 statistical software (SPSS Inc., Chicago, IL, USA) or Graphpad Prism (version 5.0; Graphpad software). The difference between two groups was determined by a two-tailed Student's *t* test. Receiver Operator Characteristic (ROC) curves and area under the ROC curve (AUC) were established to evaluate the diagnostic value of exosome miRNAs for differentiating between mTLE-HS and healthy control groups. Clinical characteristics were compared using the χ2 test of independence for qualitative variables, ANOVA or *t-test* of quantitative variables with normal distribution, the non-parametric Kruskall-Wallis test or the Mann-Whitney *U* test of quantitative variables with skewed distribution, with *P* values of < 0.05 considered to be statistically significant.

## SUPPLEMENTARY MATERIALS TABLES



## Abbreviations

mTLE-HS: mesial temporal lobe epilepsy (mTLE) with hippocampal sclerosis (HS)
AUC: area under the receiver operating characteristic curve
CI: confidence interval
GO: The Gene Ontology (GO)
KEGG: Kyoto Encyclopedia of Genes and Genomes
PCa: prostate cancer
AUC: area under the receiver operating characteristic curve
CI: confidence interval
qRT-PCR: quantitative reverse transcriptase polymerase chain reaction
GCBI: Gene-Cloud of Biotechnology Information
microRNAs: miRNAs
