# Supplementary material for: Altered microRNA profiles in plasma exosomes from mesial temporal lobe epilepsy with hippocampal sclerosis
Source: Oncotarget. 2016 Dec 1;8(3):4136–46. doi: 10.18632/oncotarget.13744 (PMC5354818; doi:10.18632/oncotarget.13744)
Supplement: Supplementary file 1 [file oncotarget-08-4136-s001.pdf]

Altered microRNA profiles in plasma exosomes from mesial temporal lobe epilepsy with hippocampal sclerosis

Supplementary Materials

Supplementary Table S1: Clinical characteristics of mTLE-HS patients and controls for miRNA microarray and qRT-PCR

Characteristic	MiRNA microarray			qRT-PCR		
	MTLE-HS (n = 3)	Controls (n = 3)	p	MTLE-HS (n = 40)	Controls (n = 40)	p
Age (mean ± SD)	28.52 ± 6.2	29.63 ± 4.8	0.9235	27.56 ± 6.3	28.72 ± 5.3	0.8690
Male/female, n.	2/1	2/1	1	25/15	22/18	0.6500

Supplementary Table S2: Demographic characteristics of patients with mTLE-HS patients and controls

Characteristic	MTLE-HS (n = 40)	Controls (n = 40)	P
Male: Female	25:15	22:18	0.6500
Age (range)	27.56 years (11–49)	28.72 years (10–50)	0.8690
Family history of epilepsy	4 (10%)	NA	NA
Course of epilepsy (range)	14.52 years (1–28)	NA	NA
Onset of seizure (range)	12.45 years (0.5–30)	NA	NA
Seizure frequency (range)(months)	35.65 (1–300)	NA	NA
Seizure time (range)	2.65 min (2 s–10 min)	NA	NA
Laterality of epileptogenic zone		NA	NA
Right	15 (40%)	NA	NA
Left	23 (50%)	NA	NA
Bilateral	2 (10%)	NA	NA
sampling time from the last seizure (range)	6.32 days (1–30)	NA	NA
AED therapy at the last clinic visit			
Valproic acid	25	NA	NA
Carbamazepine	35	NA	NA
Levetirasetam	20	NA	NA
Oxcarbazepine	26	NA	NA
Lamotrigine	10	NA	NA
Topiramate	6	NA	NA
Others	5	NA	NA
Comorbid conditions			
Hypertension	3	2	0.1817
Diabetes	2	2	
Hyperlipidemia	2	1	
Others	0	0	

NA, not applicable.
